# Actively Teaching Active Teaching Techniques

**DOI:** 10.21980/J8H94V

**Published:** 2024-10-31

**Authors:** Alice Walz, Ian Kane

**Affiliations:** * Medical University of South Carolina, Division of Pediatric Critical Care, Charleston, SC; ^ Medical University of South Carolina, Division of Pediatric Emergency Medicine, Charleston, SC

## Abstract

**Audience:**

The target audience for this small group workshop are interns and residents of any specialty.

**Introduction:**

All residents are expected to become proficient teachers in a variety of settings as they progress in training, and many residency programs offer advanced training or credentialing in medical education.^1^,^2^ Recently, some emergency medicine programs have also begun to offer a formal medical education fellowship. Traditional resident education has been in the form of didactic lectures such as morning report, noon conference, and Grand Rounds as well as small group bedside teaching by attendings. Due to the COVID-19 pandemic, in many cases these learning structures have been reengineered into a hybrid or virtual model.^3^ This new educational paradigm has spurred the search for best practice teaching methods across a variety of situations. 4 Active teaching, characterized by audience engagement and self-directed learning, has been shown to promote deeper understanding and improved knowledge retention when compared to standard didactic teaching.^5^,^6^

Educational curricula for residents now acknowledge the importance of audience participation, with more emphasis on the use of interactive teaching techniques. A review of residents-as-teachers curricula highlighted the importance of disseminating practical resources for how to effectively teach residents to be better educators.^7^ However, in the literature there are few examples of how to teach residents to implement these best practice interactive teaching methods. We designed a simple, interactive, and easily reproducible workshop for introducing the concepts of active teaching to residents that allows for active engagement with these techniques.

**Educational Objectives:**

By the end of this small group exercise, learners will be able to: 1) assess interactive teaching techniques that support learning in various environments; 2) incorporate active teaching techniques into a variety of real-world teaching scenarios; 3) implement selected techniques to enrich one’s own teaching practice.

**Educational Methods:**

Our workshop was designed to include elements of gamification, which facilitates teamwork and competition and can be used to engage learners in higher levels of learning.^8^ We began by performing a literature search for descriptions of active teaching techniques that had been used in the medical setting.^9^–^14^ We developed a list of 15 popular active teaching strategies and created a one-page menu which briefly described each strategy. Utilizing the flipped classroom model, we identified three articles (references 10, 11, and 14) which reviewed active teaching techniques and sent these articles to our participants via email one week before our session with instructions to read the articles and come prepared to discuss them at our session. We created two sets of playing cards for our activity. The first set of playing cards was titled “teaching setting” and included common venues for teaching in clinical medicine: morning report, grand rounds warm-up (which at our institution consists of a 15-minute lecture given to a large audience in a formal setting), small group, bedside teaching, journal club, and an online/virtual lecture. The second set of playing cards included simulated audiences that could be present at a teaching session: peers (residents), medical students, attendings, or mixed audience. To account for larger groups of residents, we made multiple copies of each card within the respective card set.

At the beginning of the workshop, the learning objectives were discussed and the menu with the 15 active teaching techniques was reviewed along with the assigned pre-reading articles. Residents were asked to name different active teaching techniques and give examples they experienced during their residency or medical school education. Participants (pediatric and medicine-pediatric residents) were then placed in groups of three or four, and each group drew one teaching setting card and one audience card. All groups were given the same general topic (diabetic ketoacidosis) and each group was challenged to design a short teaching activity relevant to the topic that incorporated active teaching techniques appropriate for their setting and audience. After a short period of planning, each group reported how they would teach the topic and which active teaching strategies they would use. After each group described their approach, they received peer feedback from the other groups’ participants. Key aspects of the proposed active teaching techniques and any barriers to implementation were discussed. The cards were then reshuffled for each group, and the exercise was repeated with a different general topic (in our session, we chose developmental milestones).

**Research Methods:**

Participants provided anonymous feedback in the form of surveys which assessed the efficacy of the workshop. Participants were also asked to commit to three active teaching techniques they would incorporate into teaching sessions within the next six months.

**Results:**

Our workshop was presented for two sequential years: 2022 and 2023. Our workshop was attended by 32 residents in year one and 36 residents in year two. All participants filled out the anonymous evaluation survey at the conclusion of the exercise. Eighty-five percent of respondents rated the session as “highly organized,” and a majority strongly agreed that the workshop was effective for learning active teaching techniques (78%) and taught them concrete techniques that they could incorporate into their future teaching (88%). Participants reported that they were most likely to utilize Jigsaw (31 respondents), Polling/audience response (29 respondents), case-based learning (25 respondents), role play (24 respondents) and small group activities (20 respondents) into their next teaching session. In our second workshop, there were 19 participants who had also attended the first workshop. All 19 of these residents reported using at least one active teaching technique during the previous year, and over half reported using at least three of the techniques.

**Discussion:**

Participants reported high levels of satisfaction with the organization and efficacy of this workshop. The strategies of using sets of cards to randomize the process, adding time constraints, and having each group report their teaching plans increased overall participant interest and excitement in the workshop. Having all groups design teaching sessions using the same general topic was chosen intentionally to promote friendly competition and to allow for discussion among the groups about similarities and differences in the application of their chosen active teaching techniques. To apply our activity to other specialties, we recommend choosing general topics that are commonly encountered in that specialty; for example, the topic of “myocardial infarction” or “pediatric toxidromes” could be used for emergency medicine residents.

**Topics:**

Active teaching, pediatrics, adult learning theory, graduate medical education, undergraduate medical education.

## USER GUIDE

[Table t2-jetem-9-4-sg1]

**Table t2-jetem-9-4-sg1:** 

List of Resources:	
Abstract	1
User Guide	4
Small Groups Learning Materials	8
Appendix A: Menu of Active Teaching Techniques	8
Appendix B: Print and Play Audience and Settings Cards	9
Appendix C: Post Workshop Evaluation Form	11

**Learner Audience:**

Medical Students, Interns, Junior Residents, Senior Residents

**Time Required for Implementation:**

Instructor preparation: 15 minutes, not including time spent pre-reading

Introduction/Learning objectives review: 2 minutes

Review of Active Teaching Techniques from the Handout and pre-readings: 5–15 minutes, depending on baseline resident knowledge of active teaching techniques

Group formation and selection of audience and teaching setting cards: 5 minutes

Planning session for small groups-Round 1: 5 minutes

Report from each group: 2 minutes per group

Planning session for small groups-Round 2: 5 minutes

Report from each group: 2 minutes per group

Large Group discussion of lessons learned: 5–15 minutes

**Recommended Number of Learners per Instructor**:

One Facilitator for every 3–4 small groups to ensure groups stay on task and to answer any questions from the groups during the planning sessions.

**Topics:**

Active teaching, pediatrics, adult learning theory, graduate medical education, undergraduate medical education.

**Objectives:**

By the end of this small group exercise, learners will be able to:

1. Assess interactive teaching techniques that support learning in various environments.
2. Incorporate active teaching techniques into a variety of real-world teaching scenarios.
3. Implement selected techniques to enrich one’s own teaching practice.

### Linked objectives and methods

Adult learning theory holds that learners value active involvement, reflection and questioning, and the use of previous experiences to ground and inform the acquisition of new knowledge.^4^ Curricula that use active teaching techniques are preferred by residents but have been difficult to implement, with limitations such as the difficulty of group work within a typical auditorium, frequent resident interruptions to answer pages, and faculty who fear giving up control of the learning environment by adapting their standard didactic lectures.^7^ The shift towards utilizing active teaching techniques has been made more difficult by the transition to online and asynchronous forms of education; regardless, the modern resident is expected to have a working knowledge of how to be an effective educator for a variety of audiences in a variety of settings.

We designed a simple workshop to introduce the concept of active teaching and how to utilize specific active teaching techniques in everyday practice. By making small adjustments, our workshop can easily be adapted for a variety of different learners within the field of medical education.

Educational Objective (EO) 1: Assess interactive teaching techniques that support learning in various environments. Participants were assigned pre-readings and then provided with a menu of 15 common active teaching techniques, which was reviewed prior to the start of the workshop to allow the residents to become familiar with the concepts and terminology associated with active teaching. Residents were also able to take notes on their menus and ask any clarifying questions during this discussion period.

Educational Objective (EO) 2: Incorporate active teaching techniques into a variety of real-world teaching scenarios. After the setting and audience playing cards were distributed to each group, the residents worked together to quickly develop a teaching session incorporating active teaching strategies. Since all groups were given the same overall topic, residents were able to compare the active teaching strategies chosen by the other peer groups.

Educational Objective (EO) 3: Implement selected techniques to enrich one’s own teaching practice. Our activity was designed to expose residents to common teaching scenarios they would encounter during residency and clinical practice. By choosing topics relevant to their specialty, we further attempted to illustrate that the incorporation of active teaching techniques was feasible. The survey given after our activity challenged each resident to commit to incorporating active teaching techniques into their next teaching session.

### Recommended pre-reading for facilitator

The following articles highlight active teaching techniques and theories of adult learning:

- Sivarajah RT, Curci NE, Johnson EM, et al. A review of innovative teaching methods. *Acad Radiol*. 2019;26(1):101–113. At: https://10.1016/j.acra.2018.03.025
- Wolff M, Wagner MJ, Poznanski S, Schiller J, Santen S. Not another boring lecture: engaging learners with active learning techniques*. J Emerg Med.* 2015;48(1):85–93. At: https://10.1016/j.jemermed.2014.09.010
- Steinert Y, Snell LS. Interactive lecturing: strategies for increasing participation in large group presentations. *Med Teach*. 1999;21(1): 37–42.
- Merritt C, Munzer BW, Wolff M, Santen S. Not another bedside lecture: active learning techniques for clinical instruction. *AEM Educ Train.* 2017;2(1):48–50. At: https://10.1002/aet2.10069
- Torralba KD, Doo L. Active learning strategies to improve progression from knowledge to action. *Rheum Dis Clin North Am*. 2020;46(1):1–19. At: https://10.1016/j.rdc.2019.09.001
- Mukhalalalti BA, Taylor A. Adult learning theories in context: a quick guide for healthcare professional educators. *J Med Educ Curric Dev*. 2019;6:1–10. At: https://10.1177/2382120519840332
- Thaammasitboon S, Brand PL. The physiology of learning strategies clinical teachers can adopt to facilitate learning. *Eur J Pediatr*. 2022;181(2):429–433. At: https://10.1007/s00431-021-040547

### Results and tips for successful implementation

Our activity is scheduled yearly for all residents in our program as part of their medical education curriculum. Because our participants were assigned pre-readings which reviewed the concepts of active teaching, the residents attending our session were prepared to discuss and use active teaching techniques. If our session is done without using the flipped classroom model, we recommend that residents and facilitators spend more introductory time discussing the principles of active teaching as well as examples of active teaching from the residency curriculum. Likewise, facilitators who are less familiar with the concepts of adult learning theory may find it useful to review an article which summarizes theories of adult learning in the context of medical education (pre-reading reference 6). Our session was presented in two consecutive years: 2022 and 2023. To mitigate some of the barriers seen with other active teaching initiatives, we held our session in a room with small tables and rolling chairs that facilitated the easy creation of small groups. Residents’ pagers were covered by attending providers to limit interruptions. Our activity was attended by 32 residents in year one and 36 residents in year two. Our results were obtained by surveying our participants after the completion of the activity (Table 1).

Our workshop received high marks from participants for organization and efficacy, with 85% of respondents rating the session as “highly organized.” The facilitators felt that the fast pace of the activity kept residents focused and engaged by creating an atmosphere of friendly competition. Most residents strongly agreed that the activity was effective for learning active teaching techniques (78%) and taught them concrete techniques that they could incorporate into their future teaching (88%). Residents reported that the educational value of the session was enhanced by the contributions of their peers (Table 1).

To assess which teaching techniques residents were most interested in using, we asked participants to commit to using up to three active teaching techniques within the next year. Overall, participants reported they were most likely to include Jigsaw (31 respondents), Polling/audience response (29 respondents), case-based learning (25 respondents), role play (24 respondents) and small group activities (20 respondents). The active teaching techniques participants were least likely to incorporate included thinking hats, report/debate, one-minute paper, and the muddiest point.

Slight modifications to the timing and design of the activity were made after year one in response to feedback from the residents, including decreasing the time for group planning and adding a second round of teaching session planning with a different topic. We limited groups to five minutes for planning their teaching so that we could complete two rounds within our session’s allotted time. Without this time constraint, these planning sessions could be extended to allow residents to practice other elements of curriculum design such as developing learning objectives and assessment tools. During year two we also included questions to identify residents who had completed the activity the year before; those residents were asked if they had used interactive teaching techniques during the prior year, and if so, which specific techniques. There were a total of 19 participants in our second workshop who had also attended our workshop the first year; all 19 (100%) reported using at least one active teaching technique during the previous year, and 10 of 19 (53%) reported that they used at least three different active teaching techniques during the year. Most commonly, participants self-reported using polling/audience response (14/19), case-based learning (13/19), and small group activities (11/19). Residents rarely included think-pair-share (3/19), jigsaw (2/19), or flipped classroom (1/19). Despite Jigsaw being the most common technique that residents planned to incorporate into their teaching, it was used by only two of 19 residents who had attended the previous year’s session. We hypothesize that use of the jigsaw technique may be limited by time constraints and the physical space needed for the creation of small groups. Future survey questions for residents should address specific barriers to implementation of different active teaching techniques.

### Pearls

[Table t3-jetem-9-4-sg1]

**Table t3-jetem-9-4-sg1:** 

Sample Menu of Active Teaching Techniques	
Name of technique	Definition
Role-Play	Learners act out a part or particular viewpoint to better understand the concepts and theories being discussed or to practice new skills/techniques.
Jigsaw	Topic is delivered in several smaller, interrelated pieces. Each small group is assigned a part of the topic to investigate and become expert. Groups teach their portion to the larger group to help assemble the whole puzzle. Takes a fair amount of prep by teacher, but then once rolling, the teacher is fully facilitative.
Concept Maps	Technique involves visualizing relationships between concepts by creating a diagram. Can be done individually or in groups.
Think-Pair-Share	Pose a question to group and have learners consider their response individually. Learners then pair up with neighbor to discuss and reach consensus. Then some pairs report to larger group
Polling/Audience Response	Learners anonymously commit to opinion or fact-based polls/questions. This can be used to stimulate larger conversation.
Problem Based Learning	Case based learning in small groups. Works well for management discussions that require nuance or that do not have clear guidelines.
Simulation	Allows learners to try out skills from higher stakes real life situations in a safe/low stakes setting. Works well to be linked with reflection, debrief and opportunity to try again.
Flipped Classroom	A flipped classroom is structured around the idea that lecture or direct instruction is not the best use of class time. Instead, learners encounter information before conference, freeing conference time for activities that involve higher order thinking/application of material.
Brainstorming	Brainstorming at the beginning of a session can provide an evaluation of the learner’s knowledge of a particular area prior to teaching on the topic.
Demonstration	Helpful when modeling a skill or behavior that is new for learners.
Panel Discussions	Offers multiple expert perspectives. Helpful if audience is able to submit questions in advance or have time for Q&A.
Thinking Hats	Learners wear different metaphorical hats that represent a different way of approaching a problem or topic.
Debate	Allows learners to see multiple perspectives on an issue and encourages them to listen more intently to each other before responding.
Other	

## Figures and Tables

**Table 1:** Participants’ perspectives on workshop effectiveness (N=68)

Statement	Strongly disagree	Disagree	Somewhat agree	Strongly agree
Workshop was effective for learning interactive teaching techniques	0	0	15 (22%)	53 (78%)
Feedback from peers contributed to the session’s educational value	0	0	17 (27%)	45 (73%)
I learned interactive teaching techniques that I can incorporate into my future teaching	0	0	8 (12%)	60 (88%)

## SMALL GROUPS LEARNING MATERIALS

### Appendix A Menu of Active Teaching Techniques

To be distributed at the beginning of the workshop:[Table t4-jetem-9-4-sg1]

**Table t4-jetem-9-4-sg1:**

Sample Menu of Active Teaching Techniques	
Name of technique	Definition
Role-Play	Learners act out a part or particular viewpoint to better understand the concepts and theories being discussed or to practice new skills/techniques.
Jigsaw	Topic is delivered in several smaller, interrelated pieces. Each small group is assigned a part of the topic to investigate and become expert. Groups teach their portion to the larger group to help assemble the whole puzzle. Takes a fair amount of prep by teacher, but then once rolling, the teacher is fully facilitative.
Concept Maps	Technique involves visualizing relationships between concepts by creating a diagram. Can be done individually or in groups.
Think-Pair-Share	Pose a question to group and have learners consider their response individually. Learners then pair up with neighbor to discuss and reach consensus. Then some pairs report to larger group
Polling/Audience Response	Learners anonymously commit to opinion or fact-based polls/questions. This can be used to stimulate larger conversation.
Problem Based Learning	Case based learning in small groups. Works well for management discussions that require nuance or that do not have clear guidelines.
Simulation	Allows learners to try out skills from higher stakes real life situations in a safe/low stakes setting. Works well to be linked with reflection, debrief and opportunity to try again.
Flipped Classroom	A flipped classroom is structured around the idea that lecture or direct instruction is not the best use of class time. Instead, learners encounter information before conference, freeing conference time for activities that involve higher order thinking/application of material.
Brainstorming	Brainstorming at the beginning of a session can provide an evaluation of the learner’s knowledge of a particular area prior to teaching on the topic.
Demonstration	Helpful when modeling a skill or behavior that is new for learners.
Panel Discussions	Offers multiple expert perspectives. Helpful if audience is able to submit questions in advance or have time for Q&A.
Thinking Hats	Learners wear different metaphorical hats that represent a different way of approaching a problem or topic.
Debate	Allows learners to see multiple perspectives on an issue and encourages them to listen more intently to each other before responding.
Other	

### Appendix B Print and Play Audience and Settings Cards

Print several copies and cut out individual cards. Distribute by having each group draw one audience and one setting card per round:[Table t5-jetem-9-4-sg1]

**Table t5-jetem-9-4-sg1:**

**Peers (Residents)**	**Medical Students**
**Attendings**	**Mixed Audience**
**Morning Report**	**Bedside Teaching**
**Grand Rounds Warm Up**	**Journal Club**
**Small Group**	**Online/Virtual Lecture**

### Appendix C Post Workshop Evaluation Form

[Table t6-jetem-9-4-sg1]

**Table t6-jetem-9-4-sg1:**

1. The presentation in this workshop session was:			
Poorly organized	Somewhat organized	Mostly organized	Very organized
2. The format of this workshop was an effective way to learn about different interactive teaching techniques:			
Strongly disagree	Disagree	Somewhat agree	Strongly agree
3. Receiving feedback from my peers positively contributed to the educational value of this session:			
Strongly disagree	Disagree	Somewhat agree	Strongly agree
4. I learned interactive teaching strategies that I can easily incorporate into my future teaching:			
Strongly disagree	Disagree	Somewhat agree	Strongly agree
5. After completing this workshop training, I commit to trialing the following interactive teaching techniques in the next six months (select up to three):			
Role Play	Polling/Audience Response	Flipped Classroom	Thinking Hats
One Minute Paper	Case Based Learning	Small Group Activity	Handouts
Jigsaw	Concept Maps	Simulation	The Muddiest Point
Think-Pair-Share	Problem Based Learning	Report/Debate	Other (please specify)
6. In which setting do you feel **least** comfortable teaching using active teaching techniques? (select up to three)			
Grand Rounds	Virtual/Online Lecture only		In-person + Online Lecture
Grand Rounds Warm up	Journal Club		Morning Report
Small Group	Bedside teaching		Other (please specify)
6. How can this interactive teaching workshop be improved for next time:			
